# Ortholog-Finder: A Tool for Constructing an Ortholog Data Set

**DOI:** 10.1093/gbe/evw005

**Published:** 2016-01-18

**Authors:** Tokumasa Horiike, Ryoichi Minai, Daisuke Miyata, Yoji Nakamura, Yoshio Tateno

**Affiliations:** ^1^Department of Biological and Environmental Science, Shizuoka University, Japan; ^2^The Genome Institute, Japanese Foundation of Cancer Research, Tokyo, Japan; ^3^Department of Economics, Chiba University of Commerce, Ichikawa, Japan; ^4^Research Center for Aquatic Genomics, National Research Institute of Fisheries Science, Fisheries Research Agency, Kanagawa, Japan; ^5^School of New Sciences, Daegu Gyoungbook Institute of Science and Technology, Daegu, Republic of Korea

**Keywords:** phylogenetic analysis, horizontal gene transfer, out-paralog, ortholog, prokaryote, eukaryote

## Abstract

Orthologs are widely used for phylogenetic analysis of species; however, identifying genuine orthologs among distantly related species is challenging, because genes obtained through horizontal gene transfer (HGT) and out-paralogs derived from gene duplication before speciation are often present among the predicted orthologs. We developed a program, “Ortholog-Finder,” to obtain ortholog data sets for performing phylogenetic analysis by using all open-reading frame data of species. The program includes five processes for minimizing the effects of HGT and out-paralogs in phylogeny construction: 1) HGT filtering: Genes derived from HGT could be detected and deleted from the initial sequence data set by examining their base compositions. 2) Out-paralog filtering: Out-paralogs are detected and deleted from the data set based on sequence similarity. 3) Classification of phylogenetic trees: Phylogenetic trees generated for ortholog candidates are classified as monophyletic or polyphyletic trees. 4) Tree splitting: Polyphyletic trees are bisected to obtain monophyletic trees and remove HGT genes and out-paralogs. 5) Threshold changing: Out-paralogs are further excluded from the data set based on the difference in the similarity scores of genuine orthologs and out-paralogs. We examined how out-paralogs and HGTs affected phylogenetic trees constructed for species based on ortholog data sets obtained by Ortholog-Finder with the use of simulation data, and we determined the effects of confounding factors. We then used Ortholog-Finder in phylogeny construction for 12 Gram-positive bacteria from two phyla and validated each node of the constructed tree by comparison with individually constructed ortholog trees.

## Introduction

The construction of phylogenetic trees for species plays a key role in understanding the evolution of life. In evolutionary studies, molecular phylogenetic trees are routinely constructed because phylogenetic trees of genes or genomes are expected to reflect the phylogeny of the species in question. The sequence data of homologous genes used in constructing phylogenetic trees can be readily collected by means of Basic Local Alignment Search Tool (BLAST) searches. Although the sequence data used for phylogeny construction should comprise orthologs, homologs obtained from BLAST searches are not always orthologs but rather out-paralogs of one another: They are homologs derived from gene duplication before speciation. This phenomenon causes out-paralogs to be included in the ortholog data set, and the generated phylogenetic trees are inconsistent with other trees (Koonin 2005; Snel et al. 2005). To avoid this inconsistency, phylogenetic trees have been constructed by concatenating all the orthologs used for phylogenetic analysis of species (Brown et al. 2001; Brochier et al. 2002; Wolf et al. 2002; Gadagkar et al. 2005; Ciccarelli et al. 2006). An alignment-concatenated tree allows phylogeny to be readily interpreted, because it is expected to reflect the true phylogenetic relationships of the species in question and avoid inconsistencies in the topology of the phylogenetic trees constructed for each gene. However, developing concatenated trees for orthologs including hidden out-paralogs is not a fundamental solution for obtaining accurate phylogenetic relationships. To construct a concatenated tree, ortholog data sets without out-paralogs must be prepared very carefully.

Currently, ortholog-prediction methods are classified as pairwise-species, multispecies graph-based, and multispecies tree-based methods (Trachana et al. 2011). For phylogenetic analysis of species, pairwise-species methods (Huynen and Bork 1998; Deluca et al. 2006; Ostlund et al. 2010) are unsuitable because >3 operational taxonomic units (OTUs) are required to construct a phylogenetic tree. The multispecies tree-based method (Li et al. 2006; Schneider et al. 2007; Huerta-Cepas et al. 2008) is also unsuitable for inferring the phylogeny of species because the method requires information from the species tree. Several ortholog databases have been developed using graph-based methods such as COG (Clusters of Orthologous Groups; Tatusov et al. 2000), MBGD (Uchiyama 2003), OrthoMCL-DB (Chen et al. 2006), orthoDB (Kriventseva et al. 2008), and eggNOG (Jensen et al. 2008). Although these graph-based methods are useful for predicting protein functions, they are unsuitable for phylogenetic analysis because they include out-paralogs (Trachana et al. 2011). By contrast, several stand-alone programs can be used for constructing an ortholog data set. OrthoMCL (Li et al. 2003) is one of the most widely used programs of this type, and it is based on a Markov clustering algorithm (Enright et al. 2002). DomClust—a tool for generating ortholog data sets based on the UPGMA (Unweighted Pair Group Method with Arithmetic mean), which is a hierarchical clustering algorithm, and the domain structures of the protein in use (Uchiyama 2006)—is available in the MBGD database. MultiParanoid, an extended tool of InParanoid, can identify orthology relationships between proteins in less than three species (Alexeyenko et al. 2006). Gclust, another tool used for generating an ortholog data set by means of single-linkage clustering (Sato 2009), considers the overlap score (which represents the proportion of homologous regions shared by two sequences) when clustering potential ortholog pairs into an ortholog group by using the specific *E*-value of a BLAST search. The aforementioned four programs are useful for constructing ortholog data sets that are primarily used for predicting protein function. Therefore, these programs do not aggressively exclude out-paralogs from the data set, because out-paralogs do not influence the predictions of protein function.

Out-paralogs and genes obtained through horizontal gene transfer (HGT) tend to hamper the phylogenetic analysis of species (Fitch 2000; Delsuc et al. 2005; Koonin 2005; Snel et al. 2005). To infer the relationships of 17 bacterial phyla, we previously excluded out-paralogs and HGTs from our data set through manual curation (Horiike et al. 2009). The data set enabled us to construct the phylogenetic tree with confidence, but the task was extremely complex and could not be applied to a larger data set. To overcome this problem, we developed—based on considering the aforementioned five methods—a new program, Ortholog-Finder, for constructing ortholog data sets that contain no HGT genes or out-paralogs. We examined the effects of out-paralogs and HGTs on phylogenetic trees constructed for species based on the ortholog data set obtained by Ortholog-Finder with the use of simulation data and determined the effects of the confounding factors (out-paralogs and HGT genes). We then used Ortholog-Finder to generate an ortholog data set for Actinobacteria and Firmicutes that excluded confounding genes, and thus constructed a highly reliable phylogenetic tree.

## Materials and Methods

### Implementation

Input files in the GenBank format, which includes nucleotide and amino acid sequences, or multi-FASTA format, which includes amino acid sequences, are required for each species selected. If the input files are in the GenBank format, HGT filtering is available for inferring HGT genes and removing them from the ortholog data set. If the input files are in multi-FASTA format, HGT filtering is not available because of the lack of nucleotide sequence data. To detect out-paralogs and HGT events, all chosen species must belong to one of the two taxonomic groups. Two output file formats are available for ortholog sequence data: Multi-FASTA format, which includes sequence data for each ortholog; and multi-FASTA-like format, which includes all ortholog sequence data in one file. Moreover, an alignment-concatenated tree can be constructed using the neighbor-joining (NJ) method. If users seek to construct a sequence-concatenated tree by using other programs that support the maximum-likelihood method or Bayes method, concatenated sequence data of orthologs in the phylip format can be generated by Ortholog-Finder. After HGT filtering, the inferred HGT sequences and non-HGT sequences are saved separately, and the data can be used for other analyses. Ortholog-Finder requires BLAST (Altschul et al. 1990), ClustalW (Thompson et al. 1994), MAFFT (Katoh et al. 2002), Gblocks (Castresana 2000), Bioperl (Stajich et al. 2002), EMBOSS (Rice et al. 2000), MCL (Enright et al. 2002), OrthoMCL (Li et al. 2003), and Java Runtime Environment. Ortholog-Finder operates on Linux operating systems, as tested on Ubuntu 12.04 LTS and CentOS 6.5. The program and a user guide are available at http://www.grl.shizuoka.ac.jp/∼thoriike/Ortholog-Finder.html (last accessed February 3, 2016).

### Algorithm

**Fig. 1.—:**
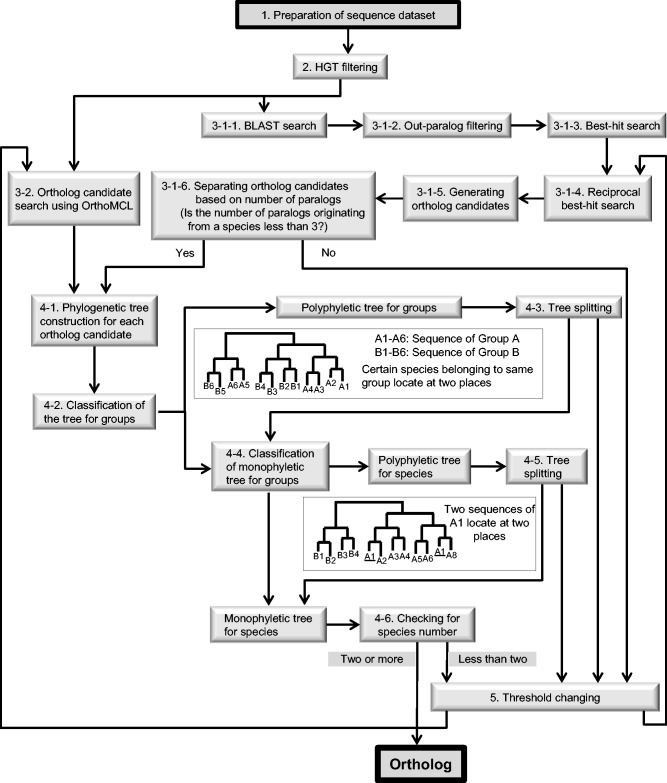
From ortholog candidate to ortholog: A flowchart.

To remove the effects of HGT genes and out-paralogs from the ortholog data set, we added five processes (HGT filtering, out-paralog filtering, classification of tree data, tree splitting, and *E*-value changing) to the conventional graph-based method of ortholog prediction. All species selected must belong to one of the two taxonomic groups because the information is required for estimating out-paralogs. The two taxonomic groups selected must be as closely related as possible, but must not be a part of the ingroup. A flowchart of the construction of ortholog data sets from sequence data is presented in figure 1.

1. Preparation of the sequence data set All open-reading frame (ORF) data, including amino acid and nucleotide data in GenBank format for a given species, must be prepared in a specific directory in the system. If users do not require HGT filtering (next paragraph), they can use amino acid sequence data in multi-FASTA format files instead of in a GenBank format file.
2. HGT filtering (optional; the default is disabled) Genes derived from HGT are inferred by the program developed by Nakamura et al. (2004). The process of HGT filtering is based on Bayesian inference performed using training models for nucleotide composition. The threshold of the chi-square test is adjustable. The default threshold is *P* = 0.005. The inferred HGT sequence data are removed from the sequence data set.
3. Finding ortholog candidates
  3-1. Identifying ortholog candidates by means of single linkage (optional; alternative step: 3-2, Markov clustering algorithm implemented using OrthoMCL)
    3-1-1. BLAST search
      All sequences are compared with each other by performing BLAST searches to detect homologs. The queries are each sequence for all organisms, and the database contains all sequence data for each organism. The default threshold is *E* = 10^−10^.
    3-1-2. Out-paralog filtering
      Out-paralog filtering is a simple process used for detecting and removing out-paralogs from the data set. It is based on the *E* value of the BLAST search result. If a hit sequence belongs to the same group as the query sequence and exhibits a lower similarity than do sequences belonging to another group, the hit sequence is considered an sout-paralog and removed from the data set (fig. 2).
    3-1-3. Best-hit search

**Fig. 2.—:**
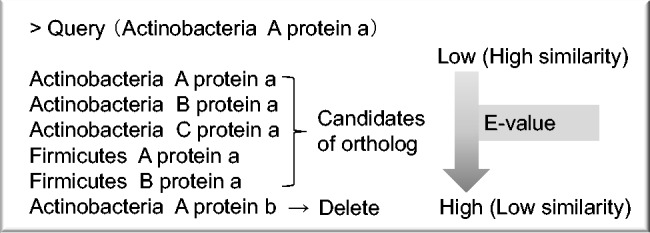
Out-paralog filtering. An example of the BLAST search is shown. The top three hit sequences are from Actinobacteria, which is the group that also contains the query sequence. The next two sequences are from Firmicutes. The sixth hit sequence is from Actinobacteria again, but the similarity is lower than that of sequences from the other group. In this case, the sixth sequence was considered an out-paralog and removed from the data set.
      The sequence in a species that is most similar to a specific sequence is obtained as the best-hit sequence. All best-hit sequences between every pair of species are detected.
    3-1-4. Reciprocal best-hit search
      Reciprocal best-hit pairs are detected as ortholog pairs based on an *E*-value between every species pair.
    3-1-5. Generating ortholog candidates
      All ortholog pairs are clustered using a single-linkage method. The clustered data are the “ortholog candidates.”
    3-1-6. Separation of ortholog candidates based on the number of paralogs
      If the number of paralogs originating from one species is ≥ 3, Steps 3-2, 4-1, 4-2, 4-3, 4-4, and 4-5 are skipped, and the ortholog candidates are advanced to Step 5. The parameter used for the number of paralogs is adjustable.
  3-2. Identifying ortholog candidates by using OrthoMCL (optional; alternative Step: 3-1, single chain clustering)
    This step of identifying ortholog candidates is an alternative to Step 3-1. OrthoMCL is based on the Markov clustering algorithm.
4. From ortholog candidate to ortholog
  4-1. Phylogenetic tree construction for each ortholog candidate
    Multiple alignments are performed for all ortholog candidates by using MAFFT (Katoh et al. 2002), and the nonconserved regions are eliminated using Gblocks (Talavera and Castresana 2007). Using the alignment data, phylogenetic trees are constructed based on the NJ method (Saitou and Nei 1987) by using ClustalW (Thompson et al. 1994).
  4-2. Classification of phylogenetic trees
    Ortholog candidate trees are constructed as unrooted trees, but the root is placed at the branch between two groups. Therefore, the trees are treated as rooted trees. Phylogenetic trees of ortholog candidates are classified as “monophyletic trees for groups” or “polyphyletic trees for groups.” Monophyletic trees are advanced to Step 4-4. Polyphyletic trees for groups contain at least two sequences that belong to the same group and share a polyphyletic relationship (an example is shown in fig. 1). In this case, at least one out-paralog or HGT is included in the ortholog candidate data, and the data are advanced to Step 4-3.
  4-3. Tree splitting of polyphyletic trees for groups (optional; the default is enabled)
    Tree splitting is a novel method used for removing out-paralogs from the ortholog candidates. The concept of tree splitting is described in figure 3. In the tree-splitting procedure, branch-cutting is attempted in single steps—starting from the longest branch and then proceeding to the next longest uncut branch if the first attempt is unsuccessful, and so on—until a monophyletic tree is obtained. If the monophyletic tree for groups contains at least one species for each group, the tree data are advanced to Step 4-4. If a monophyletic tree cannot be obtained, the data are restored and advanced to Step 5.
  4-4. Classification of polyphyletic trees for groups

**Fig. 3.—:**
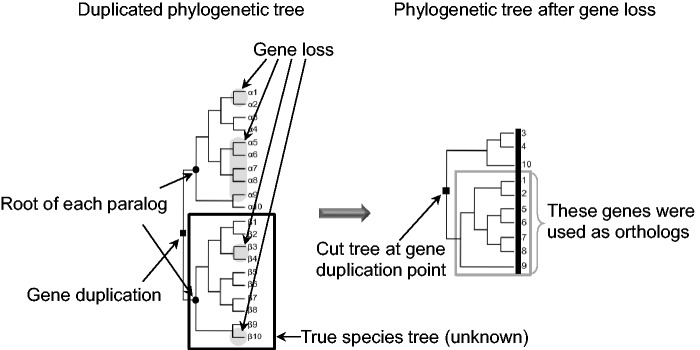
Example of tree splitting for gene loss after gene duplication. The tree on the left is a duplicated phylogenetic tree from the common ancestor. This tree contains two lineages (a group containing Species 1–8 and one containing Species 9 and 10). After gene duplication, the alpha and beta genes appeared; these genes diverged because of species divergence. Subsequently, gene losses occurred in the lineages that are displayed using a shadow. The tree on the right was constructed using the existing genes. Information on the paralogs alpha and beta was lost (shown in the black bar), and thus the paralogs were not recognized. Here, the topology of the tree on the right is not consistent with the species tree indicated with a black frame on the tree on the left. Therefore, the tree on the right should not be used as an ortholog tree. However, in the case where information was available on the position of outgroups (Species 9 and 10 form an outgroup of the Species 1–8 group), the gene-duplication point shown in the closed square can be inferred. When the tree on the right is bisected at the duplication point, two trees are obtained, and because one of these trees now contains few species, this segment is eliminated. The topology of the other tree, displayed in a gray frame, is consistent with a species tree. Consequently, the OTUs in the tree shown in the gray frame are available as orthologs.
    These trees are classified as “monophyletic tree for species” or “polyphyletic tree for species.” The OTUs in the monophyletic tree are advanced to Step 4-6. Polyphyletic trees for species contain at least two sequences that are from the same species and share a polyphyletic relationship (an example is shown in fig. 1). In this case, at least one out-paralog or HGT is included in the ortholog candidate data set, and the data are advanced to Step 4-5.
  4-5. Tree splitting of polyphyletic trees for species (optional; the default is enabled)
    This step is almost identical to Step 4-3. Here, branch-cutting is attempted in single steps starting from the longest branch until the tree contains at most one gene per species. If the monophyletic tree for species contains at least one species for each group, the OTUs in the monophyletic tree are advanced to Step 4-6. If a monophyletic tree cannot be obtained, the data are restored and advanced to Step 5.
  4-6. Checking for species number
    Users can set the minimum number of species for each group in an ortholog data set. The default value is 2. If the number of species for a group is less than the minimum number, the data are not added to the ortholog data set and are advanced to Step 5. If the number of species is ≥ 2, the OTUs in the monophyletic tree are judged to be orthologs and are added to the ortholog data set.
5. Threshold changing All data are returned to Step 3-1-3 (best-hit search) and the threshold is changed. This process is cycled with the threshold (BLAST *E* value) being changed from 10^−10^ to 10^−100^ at a 10^−10^ decrement. In theory, the threshold for homology must be set appropriately to detect orthologs, because the sequence similarities of each protein family depend on evolutionary rates. Therefore, at the first cycle, Ortholog-Finder uses a loose threshold (*E* value = 10^−10^) for proteins that evolve rapidly. At the next cycle, a stricter threshold than the first one is used and orthologs are detected in protein families that evolve slowly. The concept of threshold changing is depicted in figure 4. Users can set the thresholds.
6. Phylogenetic tree construction

**Fig. 4.—:**
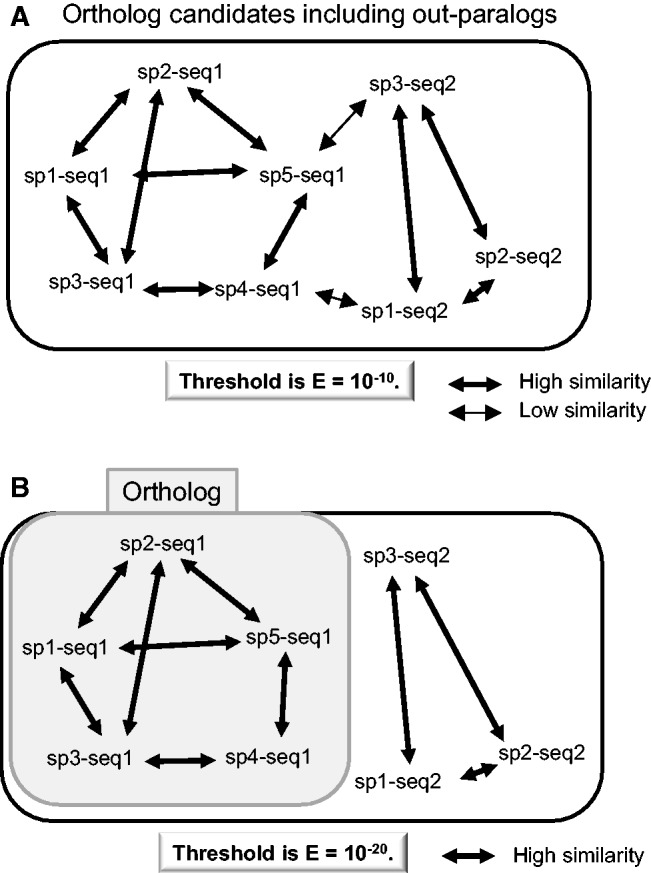
Example of threshold changing. These graphs show the linkage of proteins based on similarity. The *sp* number represents each species, and the *seq* number represents each protein. Broad and narrow arrows indicate high and low similarities, respectively. In cases of *E* = 10^−10^, an ortholog cannot be determined, because out-paralogs link to the genuine ortholog as a part of the ortholog candidate (*A*). When the threshold becomes more strict (*E* = 10^−20^), the linkage between genuine orthologs and out-paralogs breaks, and the genuine orthologs are detected (*B*). In this case, the proteins of sp1–seq2, sp2–seq2, and sp3–seq2 cannot be detected as orthologs because the number of species is insufficient (see Materials and Methods).

Ortholog-Finder can construct a concatenated tree by using the ortholog data set and the NJ method. Multiple alignments are performed for each ortholog by using MAFFT, and the nonconserved regions in the alignment are eliminated using Gblocks. The alignment data are concatenated into one alignment data set. The orthologs do not always include all species. If certain multiple-alignment data sets lack several species, gaps are inserted at the site of the missing data in the concatenated multiple alignment. Using a large amount of alignment data, a concatenated tree is constructed using ClustalW, based on the NJ method. Phylogenetic trees for each ortholog are constructed using the NJ method in order to calculate the branch-support percentage, which reflects the percentage of ortholog trees that exhibits the same branching pattern as the concatenated tree, and this is calculated at each internal branch. If a branch of a concatenated tree is shared with an internal branch of several orthologs, the branch-support percentage will be high (i.e., the reliability of the branch will be high). Furthermore, the bootstrap test is performed to illustrate the reliability of each internal branch of the concatenated tree. Ortholog-Finder does not support the maximum-likelihood method or Bayes method because the calculation times required for choosing the optimal substitution model and constructing phylogenetic trees are extremely long. For users who wish to use these methods, Ortholog-Finder provides concatenated sequence data of orthologs in the phylip format and the data can be used in another tree-building program.

### Simulations for Gene Loss after Gene Duplication

To evaluate the quality of the ortholog data obtained using Ortholog-Finder and the phylogenetic tree constructed based on the ortholog data, we tested them by using simulated data under several conditions. We began by testing the effect of out-paralogs on the generation of ortholog data and the phylogenetic analysis, because out-paralogs hamper the estimation of species phylogeny (Fitch 2000; Delsuc et al. 2005; Koonin 2005; Snel et al. 2005). We performed the simulation test by using “Ortholog-Finder with single linkage” (OF-S; *Algorithm* 3-1), “Ortholog-Finder with OrthoMCL” (OF-M; *Algorithm* 3-2), and OrthoMCL alone (OM), as discussed in the following paragraph.

First, we generated a tree featuring 32 OTUs as the model tree. The lengths of branches between neighboring OTUs or nodes were 0.04. To generate simulation data containing out-paralogs, the model tree was duplicated to simulate the gene-duplication event of the common ancestor, and this yielded 64 OTUs. Branch lengths between the roots of two duplicates were set to 0.02, 0.04, or 0.08, and each condition was tested. The simulated amino acid sequences, which contained 200 amino acids corresponding to each OTU in the duplicated tree, were generated by using indel-Seq-Gen Ver. 2.01.03 (Strope et al. 2007) with default parameters. To simulate gene losses, certain percentages of genes among the ortholog candidates were randomly deleted (30–85% deleted, at 5% increments). The sequence data of 100 homologs were generated by the simulations, after which we removed the information on individual homologs by sorting sequences according to species when testing the performance of Ortholog-Finder in the construction of ortholog data sets. Using Ortholog-Finder, ortholog groups were identified and the ortholog-sequence-concatenated trees were constructed using the NJ method. All parameters used with Ortholog-Finder were the defaults (default parameters in *Algorithm*). One hundred concatenated trees were constructed by running 100 trials, and, using the NJ method, phylogenetic trees were also constructed for each of the orthologs that were generated by the 100 trials. To evaluate the quality of the ortholog data set generated by Ortholog-Finder, we calculated the accuracy rates of the concatenated trees (percentage of ortholog-sequence-concatenated trees exhibiting the same topology as the model tree) and each ortholog tree (percentage of ortholog trees exhibiting the same topology as the model tree) at each tested branch length and gene-loss percentage.

We calculated the accuracy rates for the simulated data generated not only by OF-S (as noted in the previous paragraph) but also by OF-M, which uses an external OrthoMCL program to cluster amino acid sequences as candidates of orthologs based on a Markov clustering algorithm. To compare the quality of the ortholog data generated by Ortholog-Finder with that of the data generated by OrthoMCL, we calculated the accuracy rates of the ortholog-sequence-concatenated trees and those of the ortholog trees constructed using the ortholog data generated by OM. The BLAST threshold used in OrthoMCL was *E* = 10^−10^.

### Simulations for HGT

We tested the influence of HGT on ortholog data and the phylogenetic analysis by using simulated sequence data. The model tree was a simple bifurcated tree featuring 32 OTUs. The branch lengths between neighboring OTUs or nodes were 0.04. We simulated HGT events on the model tree at random lineages to generate “horizontally gene-transferred trees” or “HGT trees” (Horiike et al. 2011). We assumed that HGT occurrence follows Poisson distribution, and HGT trees were constructed using the parameter mean HGT frequency (0.0–5.0; see Results and Discussion). Amino acid sequence data based on the HGT trees were generated using indel-Seq-Gen. The original sequence contained 200 amino acids and all other parameters were defaults. After the simulated ortholog sequence data were generated, we removed the information on individual orthologs by sorting the sequences according to species when testing the performance of Ortholog-Finder in identifying orthologs. Using Ortholog-Finder, ortholog groups were identified once again and the ortholog-sequence-concatenated trees were constructed using the NJ method. All parameters of Ortholog-Finder were the defaults. Phylogenetic trees for each ortholog were also constructed using the NJ method. To evaluate the quality of the ortholog data sets obtained by Ortholog-Finder, the accuracy rates of concatenated trees and each ortholog tree were calculated at each HGT frequency.

We calculated the accuracy rates for the simulated data generated by not only OF-S but also OF-M and OM; the calculation was exactly as described in the last paragraph of the previous subsection.

### Simulations for Long Evolutionary Distance

We tested Ortholog-Finder by using sequences that were increasingly more divergent (branch lengths were increased until the sequences were in saturation). The model tree was a simple bifurcated tree featuring 32 OTUs. The branch lengths between neighboring OTUs or nodes were set to 0.02, 0.04, 0.06, 0.08, 0.10, 0.12, 0.14, 0.16, 0.18, 0.20, 0.22, or 0.24. Amino acid sequence data based on the trees were generated using indel-Seq-Gen. The processes of the simulation test after sequence generation were same as those of “Simulations for HGT.”

### Phylogenetic Analysis for 12 Gram-Positive Bacteria

We used Ortholog-Finder to analyze the phylogeny of 12 Gram-positive bacteria for which genome data were available (table 1). The 12 bacteria belong to two phyla, Actinobacteria (five species from distinct families) and Firmicutes (seven species from distinct families), and therefore these could be used as outgroups for each other. The protein sequences, translated from predicted ORFs, were obtained from the NCBI site ftp://ftp.ncbi.nlm.nih.gov. Ortholog-Finder parameters were set to the defaults, with the exception being that HGT filtering was enabled. The ortholog-sequence-concatenated tree was constructed according to the NJ method by using Ortholog-Finder. Bootstrap values and branch-support percentages were calculated to examine the reliability of the internal branches of the concatenated tree.

**Table 1:** List of Organisms

Phylum	Species
Actinobacteria	*Acidimicrobium ferrooxidans* (DSM 10331)
	*Bifidobacterium longum* (NCC2705)
	*Cryptobacterium curtum* (DSM 15641)
	*Mycobacterium tuberculosis* (H37Rv)
	*Rubrobacter xylanophilus* (DSM 9941)
Firmicutes	*Bacillus subtilis* (168)
	*Clostridium acetobutylicum* (ATCC 824)
	*Halothermothrix orenii* (H 168)
	*Natranaerobius thermophilus* (JW/NM-WN-LF)
	*Staphylococcus aureus* (N315)
	*Streptococcus pyogenes* (SF370)
	*Thermoanaerobacter tengcongensis* (MB4T)

The ortholog-sequence-concatenated tree created based on the maximum-likelihood method was constructed using RAxML Version 8.0.20 (Stamatakis et al. 2005) in order to compare the phylogenetic tree shape with the shape of the tree generated using the NJ method. The substitution model and the parameters were estimated automatically by RAxML.

## Results and Discussion

### Simulations for Gene Loss after Gene Duplication

To test the performance of Ortholog-Finder against out-paralogs, we calculated the accuracy rate of ortholog-sequence-concatenated trees, which revealed the percentage of concatenated trees that exhibited the accurate topology. Because we assumed that out-paralogs in ortholog data are generated by gene loss after gene duplication, the model trees were duplicated in the common ancestor, and certain genes were randomly deleted from the ortholog candidates to simulate gene-loss events. Simulation tests were performed using OF-S, OF-M, and OM. OrthoMCL is a program used for constructing ortholog data sets (see Introduction). In OF-M, Ortholog-Finder uses OrthoMCL to cluster sequences by employing a Markov clustering algorithm (see Materials and Methods). The accuracy rate allows investigators to examine the influence of out-paralogs generated as a result of gene loss after gene duplication (fig. 5). When the branch length between the two duplicated trees was 0.02 (fig. 5*A*), most trees were accurately reproduced when gene-loss percentage was <40%. However, the accuracy of trees generated by OM gradually decreased when the gene-loss percentage was >40%. The accuracies of the remaining trees (OF-S, OF-M) decreased sharply starting from a gene-loss percentage of 70%. When the branch length between the two duplicated trees was 0.04 (fig. 5*B*), most of the trees were accurately reproduced when the gene-loss percentage was <45%. The accuracy of trees generated by OM decreased starting from 45% gene loss, whereas the accuracies of the other trees (OF-S, OF-M) decreased sharply starting from 75% gene loss. At 80% gene loss, the accuracy rate of the tree generated based on the Markov clustering algorithm (OF-M) was greater than that of the tree generated by means of single linkage (OF-S). When the branch length between the roots of two duplicated trees was 0.08 (fig. 5*C*), most of the trees were accurately reproduced when the gene-loss percentage was <45%. The accuracy of the trees generated by OM and OF-S declined rapidly starting from 45% and 80% gene loss, respectively, but that of the tree generated by OF-M did not decrease markedly. When the gene-loss percentage was 85%, the accuracy rates of all trees were 0%. We also calculated the accuracy rate of the phylogenetic tree of each ortholog, which showed the percentage of ortholog trees that exhibited the accurate topology for each tested branch length between the roots: 0.02, 0.04, and 0.08 (fig. 5*D–F*). The accuracy rates measured for OM decreased sharply at 60% or 65% gene loss, but those measured for OF-S or OF-M gradually decreased when the branch lengths were 0.02, 0.04, and 0.08. These results reveal five key points: 1) When the gene-loss percentage was <45%, the model tree was accurately reconstructed under all conditions; 2) when the gene-loss percentage was >45%, the accuracy rates of the tree generated by Ortholog-Finder were greater than those of the tree generated by OM; 3) when the gene-loss percentage was 75% or 80%, accuracy decreased drastically; 4) when the branch length between the duplicated trees was increased, the accuracy rates also increased at the same gene-loss percentage; and 5) when gene-loss percentage was <60%, most of the ortholog trees generated by OF-S, OF-M, and OM were consistent with the model tree; however, when the gene-loss percentage was >60%, the accuracy rate of only the ortholog tree generated by OM decreased sharply.
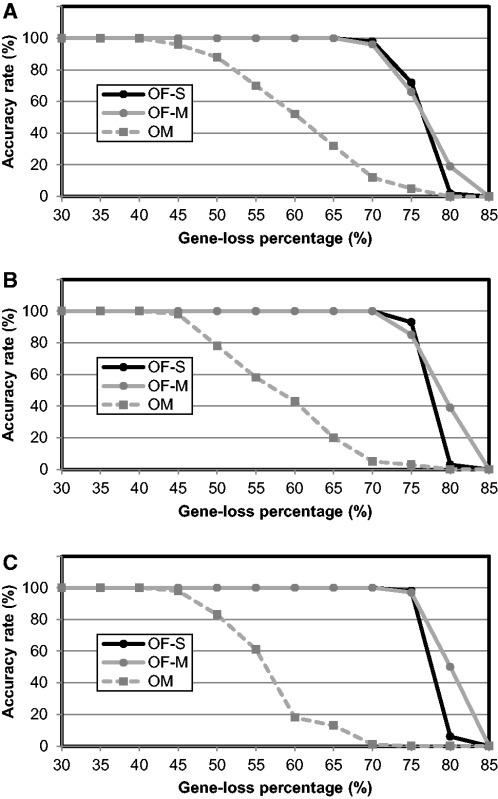

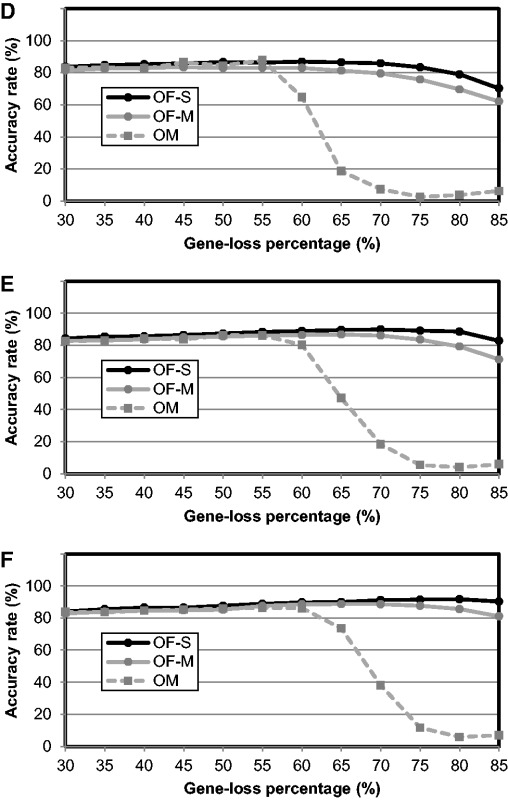

**Fig. 5.—:** Accuracy rates of the phylogenetic tree constructed for out-paralog simulation data. Accuracy rates of the phylogenetic trees were calculated at various gene-loss percentages. OF-S (black circles), OF-M (gray circles), and OM (gray squares): OrthoMCL. Accuracy rates of the sequence-concatenated tree when the branch lengths between the roots of two duplicates were 0.02 (*A*), 0.04 (*B*), and 0.08 (*C*). Accuracy rates of the tree of individual orthologs when the branch lengths between the roots of two duplicates were 0.02 (*D*), 0.04 (*E*), and 0.08 (*F*).

### Simulations for HGT

**Fig. 6.—:**
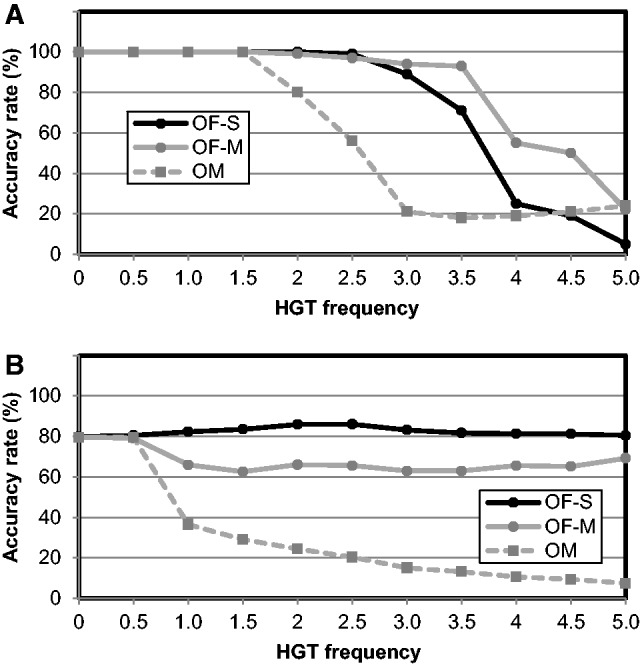
Accuracy rates of the phylogenetic tree constructed for the HGT simulation data. Accuracy rates of the phylogenetic trees were calculated at various HGT frequencies. OF-S: black circles; OF-M: gray circles; OM: gray squares.

To test the performance of Ortholog-Finder against HGT, we calculated the accuracy rate (%) by conducting computer simulations. A bifurcated tree featuring 32 OTUs was used as a model tree. Because we assumed that HGT occurrence follows a Poisson distribution, HGT trees were constructed using the parameter of mean HGT frequency (0.0, 0.5, 1.0, 1.5, 2.0, 2.5, 3.0, 3.5, 4.0, 4.5, and 5.0). Amino acid sequence data corresponding to the HGT tree were generated using indel-Seq-Gen. When the mean HGT frequency was ≤1.5, the model tree was perfectly reproduced by OF-S, OF-M, and OM (fig. 6). When the mean HGT frequency was ≤3, the model tree was mostly reproduced, with the exception being the case of the ortholog data generated by OM (fig. 6). When the mean HGT frequency was >3, the accuracy rates of the trees generated by OF-S and OF-M decreased with an increase in HGT frequency. When the mean HGT frequency was 5, an accurate tree could not be readily constructed by OF-S, OF-M, or OM. These results suggest that Ortholog-Finder (OF-M and OF-S) can tolerate HGT events, because most of the phylogenetic trees were accurately reproduced even when the mean HGT frequency was 3. Thus, even if HGT events occur greater than three times, HGT-filter (see Materials and Methods) removes some of the HGT genes and reduces the effect of HGT.

We calculated the accuracy rates of the phylogenetic trees constructed for each ortholog. When mean HGT frequency was ≤0.5, the accuracy rates of ortholog trees generated by OF-S, OF-M, and OM were all approximately 80%. The accuracy rate of ortholog trees generated by OF-S stayed around 80%, but that of the trees generated by OF-M decreased to around 65%; the accuracy rate of the ortholog tree generated by OM decreased to < 10%.

### Simulations for Long Evolutionary Distance

**Fig. 7.—:**
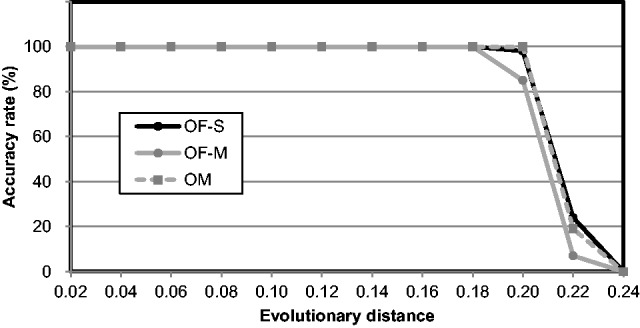
Accuracy rates of the phylogenetic tree constructed for the evolutionary-distance simulation data. Accuracy rates of the phylogenetic trees were calculated at various evolutionary distances. OF-S: black circles; OF-M: gray circles; and OM: gray squares.

To test the performance of Ortholog-Finder in a near-saturation scenario, we calculated the accuracy rate (%) by conducting computer simulations. The accuracy rates for the tree featuring distinct branch lengths (0.02–0.24, 0.02 increment) are shown in figure 7. When the branch length ranged from 0.02 to 0.18, the phylogenetic trees were accurately generated by OF-S, OF-M, and OM. The accuracy rate of the tree generated by OF-M decreased starting from a branch length of 0.18, and from a branch length of 0.20, the accuracies of all trees (OF-S, OF-M, OM) decreased sharply. When the branch length was 0.24, the accuracy rates of all trees were 0%. The effects of saturation on OF-S, OF-M, and OM were almost identical. Thus, the genuine tree cannot be readily reproduced by constructing concatenated trees by using orthologs.

### Phylogenetic Analysis of Gram-Positive Bacteria

We used the sequence data of 12 Gram-positive bacteria (table 1) and created the ortholog data set by using OF-M and constructed the sequence-concatenated tree by using the NJ method. We first obtained 843 sets of ortholog candidates after implementation of the Markov clustering algorithm by OrthoMCL (see *Algorithm* and fig. 1). When the threshold was *E* = 10^−10^, 425 trees were identified as monophyletic trees (table 2). After tree spitting, 188 polyphyletic trees became monophyletic trees and they were joined as orthologs. In total, 613 trees were obtained as orthologs when the threshold was reached at the first cycle (*E* = 10^−10^). Furthermore, 65 orthologs were obtained through threshold changing, and 56 orthologs were generated by applying the threshold-changing and tree-splitting operations. In the end, 734 orthologs were generated by Ortholog-Finder.

**Table 2:** Number of Phylogenetic Trees and Orthologs Generated by OF-M under Each Tested Threshold

*E* Value	Monophyletic Trees	Monophyletic Trees after Tree Splitting	orthologs
10^−10^	425	188	613
10^−20^	25	22	47
10^−30^	13	10	23
10^−40^	9	9	18
10^−50^	8	8	16
10^−60^	4	3	7
10^−70^	3	1	4
10^−80^	1	2	3
10^−90^	1	0	1
10^−100^	1	1	2
Total	490	244	734

**Fig. 8.—:**
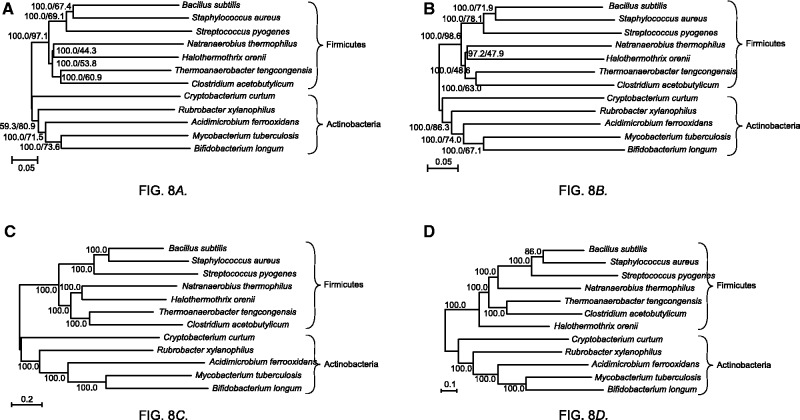
Phylogenetic tree constructed for 12 Gram-positive bacteria. A phylogenetic tree for 12 Gram-positive bacteria was constructed based on the NJ method and using the ortholog data set generated by OF-M (*A*) and OF-S (*B*). The bootstrap values and the branch-support percentage for each internal branch are displayed on left and right, respectively. A phylogenetic tree for the 12 Gram-positive bacteria was constructed based on the maximum-likelihood method and using the ortholog data set generated by OF-M (*C*) and OF-S (*D*).

The 734 orthologs obtained were used for constructing the concatenated tree for Actinobacteria and Firmicutes (fig. 8*A*). The bootstrap values on each internal branch were 100%. This result indicates that the bootstrap test may not be adequate for testing the reliability of each internal branch of the concatenated tree because the various reliabilities of each internal branch were not sufficiently estimated (Salichos and Rokas 2013). The “branch-support percentage” independently reflects the number of orthologs that support the branching pattern for each internal branch on the concatenated tree. The branch-support percentages indicated that the support percentage of short branches in the Firmicutes clade tended to be low. Therefore, we caution users that short branches in a tree indicate low reliability, and thus biological inferences made from such a tree might be suspect.

Similarly, 146 orthologs were obtained by OF-S, and the concatenated phylogenetic tree was constructed using the NJ method (fig. 8*B*); the shape of this tree is identical to that of the tree generated by OF-M. The number of orthologs obtained using OF-M (734) and OF-S (146) differed substantially because of the difference in the algorithms used for finding orthologs. In OF-S, we selected reciprocal best hit for single linkage in order to avoid false-positive orthologs in our phylogenetic analysis. However, the generated phylogenetic trees show that the orthologs obtained using OrthoMCL (OF-M) are also sufficient for phylogenetic analysis.

We constructed sequence-concatenated trees by using the maximum-likelihood method; we used RAxML Version 8.0.20 and the ortholog data sets generated by OF-M and OF-S (fig. 8*C and D*). The maximum-likelihood tree constructed using the ortholog data generated by OF-M was identical in topology to the two NJ concatenated trees generated by OF-M and OF-S. The topologies of the three concatenated trees are more reliable than the topology of the maximum-likelihood tree generated by OF-S. A part of the maximum-likelihood tree generated by OF-S differed slightly from the other trees, but most of the branching patterns in the tree were the same as those in the other concatenated trees.

### Removing Out-Paralog and HGT Genes

The existence of in-paralogs in an ortholog data set does not generally affect the estimation of the species phylogenetic tree. Therefore, if > 1 in-paralog is included among the ortholog candidates, one of the in-paralogs is randomly selected to obtain the ortholog data set. Out-paralogs generated before the divergence of two groups are removed by means of out-paralog filtering, tree splitting, and threshold changing. Conversely, out-paralogs generated after the divergence of two groups are not removed by out-paralog filtering and threshold changing, but tree splitting is useful for removing the out-paralogs. By using HGT filtering, HGTs within and between groups are removed from the data set, and some of the remaining HGTs are removed through tree splitting. If the timespan between gene duplication and speciation is extremely short, identifying in/out-paralogs is challenging. However, the bootstrap value or the branch-support percentage of internal branches of the concatenated tree indirectly shows the quality of an ortholog data set.

## Limitations of Ortholog-Finder

Certain limitations of Ortholog-Finder are the following. 1) To obtain orthologs, Ortholog-Finder requires two known groups of species, because the program identifies and removes out-paralogs based on the phylogenetic tree constructed with a root. Therefore, if a user plans to use only one group of species, another group of species must be added as an outgroup. 2) Most of the run time of Ortholog-Finder is related to the HGT filter, which requires a long calculation time. If a user selects an extremely large amount of species data to use with Ortholog-Finder, a sequence data set without HGT must be prepared for each species. Users can remove HGT genes from the sequence data set generated by Ortholog-Finder by selecting the option “HGT filtering only” in advance. 3) NJ trees are recognized to feature long branches and suffer from long-branch attraction. Therefore, certain orthologs affected by such trees might be included in the ortholog database. 4) We were able to use Ortholog-Finder and obtain ortholog data for eukaryotes, but an extremely long time was required for completing the process (e.g., 2 weeks for 12 species); the results obtained for mammals are presented in supplementary material, Supplementary Material online. We believe that our program can be used for eukaryotes, but the number of species that can be included is limited.

## Conclusion

In this study, we developed Ortholog-Finder, a program for constructing ortholog data sets for phylogenetic analysis. To eliminate the effects of HGT genes and out-paralogs from the ortholog data set, this program includes five processes: HGT filtering, out-paralog filtering, classification of tree data, tree splitting, and *E*-value changing. We examined the effects of out-paralogs and HGTs on phylogenetic trees constructed for species based on the ortholog data set obtained by Ortholog-Finder with the use of simulation data, and we determined the effects of confounding factors. Our results suggest that Ortholog-Finder can tolerate gene loss after gene duplication and HGT events, because most of the phylogenetic trees were accurately reproduced even when these events occurred. Finally, we used Ortholog-Finder to generate an ortholog data set for 12 Gram-positive bacteria (Actinobacteria and Firmicutes) and validated each node of the constructed tree by comparison with individually constructed ortholog trees.

## Supplementary Material

Supplementary figure S1 and tables S1 and S2 are available at *Genome Biology and Evolution* online (http://www.gbe.oxfordjournals.org/).

Supplementary Data
