# Supplementary material for: Ortholog-Finder: A Tool for Constructing an Ortholog Data Set
Source: Genome Biol Evol. 2016 Jan 18;8(2):446–57. doi: 10.1093/gbe/evw005 (PMC4779612; doi:10.1093/gbe/evw005)
Supplement: Supplementary Data [file supp_8_2_446__index.html]

Ortholog-Finder: A Tool for Constructing an Ortholog Data Set — Supplementary Data 

# Ortholog-Finder: A Tool for Constructing an Ortholog Data Set

## Supplementary Data

files

- Supplementary Data - zip file
