# Supplementary material for: Ortholog-Finder: A Tool for Constructing an Ortholog Data Set
Source: Genome Biol Evol. 2016 Jan 18;8(2):446–57. doi: 10.1093/gbe/evw005 (PMC4779612; doi:10.1093/gbe/evw005)
Supplement: Supplementary Data [file supp_evw005_suppl_data.zip › SupplementaryTable2.pdf]

Supplementary table S2. Number of phylogenetic trees for 12 mammals and the orthologs generated by OF-S under each tested threshold.

E-value	Monophyletic trees	Monophyletic trees after tree splitting	orthologs
10^{-10}	8150	390	8540
10^{-20}	3	0	3
10^{-30}	0	0	0
10^{-40}	2	3	5
10^{-50}	0	1	1
10^{-60}	0	1	1
10^{-70}	1	0	1
10^{-80}	3	1	4
10^{-90}	3	0	3
10^{-100}	1	2	3
Total	8163	398	8561
