# Supplementary material for: Ortholog-Finder: A Tool for Constructing an Ortholog Data Set
Source: Genome Biol Evol. 2016 Jan 18;8(2):446–57. doi: 10.1093/gbe/evw005 (PMC4779612; doi:10.1093/gbe/evw005)
Supplement: Supplementary Data [file supp_evw005_suppl_data.zip › Horiike_supplementary_manuscript1009.docx]

**Supplementary Data**

**Phylogenetic analysis for 12 mammals**

**Methods**

We used Ortholog-Finder to analyze the phylogeny of 12 mammals for which genome data were available (supplementary table S1). The 12 mammals belong to 2 groups, Metatheria (2 species) and Eutheria (10 species). The protein sequences, translated from predicted ORFs, were obtained from the NCBI site ftp://ftp.ncbi.nlm.nih.gov. Ortholog-Finder parameters were set to the defaults. The ortholog-sequence-concatenated tree was constructed according to the NJ method by using Ortholog-Finder. Bootstrap values and branch-support percentages were calculated to examine the reliability of the internal branches of the concatenated tree.

The ortholog-sequence-concatenated tree created based on the maximum likelihood method was constructed using RAxML Version 8.0.20 (Stamatakis et al. 2005) in order to compare the phylogenetic tree shape with the shape of the tree generated using the NJ method. The substitution model and the parameters were estimated automatically by RAxML.

**Results and Discussion**

We used the sequence data of 12 mammals (supplementary table S1) and created the ortholog dataset by using OF-S and constructed the sequence-concatenated tree by using the NJ method. When the threshold was E = 10^-10^, 8150 trees were identified as monophyletic trees. After tree spitting, 390 polyphyletic trees became monophyletic trees and they were joined as orthologs. In total, 8540 trees were obtained as orthologs when the threshold was reached at the first cycle (E = 10^-10^). Furthermore, 13 orthologs were obtained through threshold changing, and 8 orthologs were generated by applying the threshold-changing and tree-splitting operations. In the end, 8561 orthologs were generated by Ortholog-Finder (supplementary table S2).

The 8561 orthologs obtained were used for constructing the concatenated tree for Metatheria and Eutheria (supplementary fig. S1A). The bootstrap values on each internal branch were 100%. The branch-support percentages indicated that the support percentage of short branches in the Eutheria clade tended to be low. We constructed sequence-concatenated trees by using the maximum likelihood method; we used RAxML Version 8.0.20 and the ortholog datasets generated by OF-S (supplementary fig. S1B). The bootstrap values on each internal branch were 100%, except for 94% in the case of an internal branch that separates *Dasypus novemcinctus* (Xenarthra) and other Eutheria (Laurasiatheria, Euarchontoglires, and Afrotheria). The phylogenetic position of *D. novemcinctus* differed in the maximum likelihood tree and the NJ concatenated trees. The phylogenetic position of *D. novemcinctus* (Xenarthra) has remained unclear because the common placental ancestor diverged into Boreotheria (Laurasiatheria and Euarchontoglires), Afrotheria, and Xenarthra nearly simultaneously (Nishihara et al. 2009).

**Figure Legend**

SUPPLEMENTARY FIG. S1. Phylogenetic tree constructed for 12 mammals.

A phylogenetic tree for 12 mammals was constructed using the ortholog dataset generated by OF-S and based on the (*A*) NJ method and (*B*) the maximum likelihood method. In *A*, the bootstrap values and the branch-support percentage for each internal branch are displayed on the left and right of “/,” respectively.
