# Supplementary material for: Ortholog-Finder: A Tool for Constructing an Ortholog Data Set
Source: Genome Biol Evol. 2016 Jan 18;8(2):446–57. doi: 10.1093/gbe/evw005 (PMC4779612; doi:10.1093/gbe/evw005)
Supplement: Supplementary Data [file supp_evw005_suppl_data.zip › SupplementaryTable1.pdf]

Supplementary table S1. List of organisms.

Subclass	Superorder	Species
Metatheria	Marsupialia	*Monodelphis domestica*
	Marsupialia	*Sarcophilus harrisii*
Eutheria	Laurasiatheria	*Canis familiaris*
	Laurasiatheria	*Odobenus rosmarus*
	Laurasiatheria	*Bos taurus*
	Laurasiatheria	*Tursiops truncatus*
	Laurasiatheria	*Orcinus orca*
	Euarchontoglires	*Homo sapiens*
	Euarchontoglires	*Pan troglodytes*
	Afrotheria	*Trichechus manatus*
	Afrotheria	*Loxodonta africans*
	Xenarthra	*Dasypus novemcinctus*
