# Supplementary figures and images for: Ortholog-Finder: A Tool for Constructing an Ortholog Data Set
Source: Genome Biol Evol. 2016 Jan 18;8(2):446–57. doi: 10.1093/gbe/evw005 (PMC4779612; doi:10.1093/gbe/evw005)

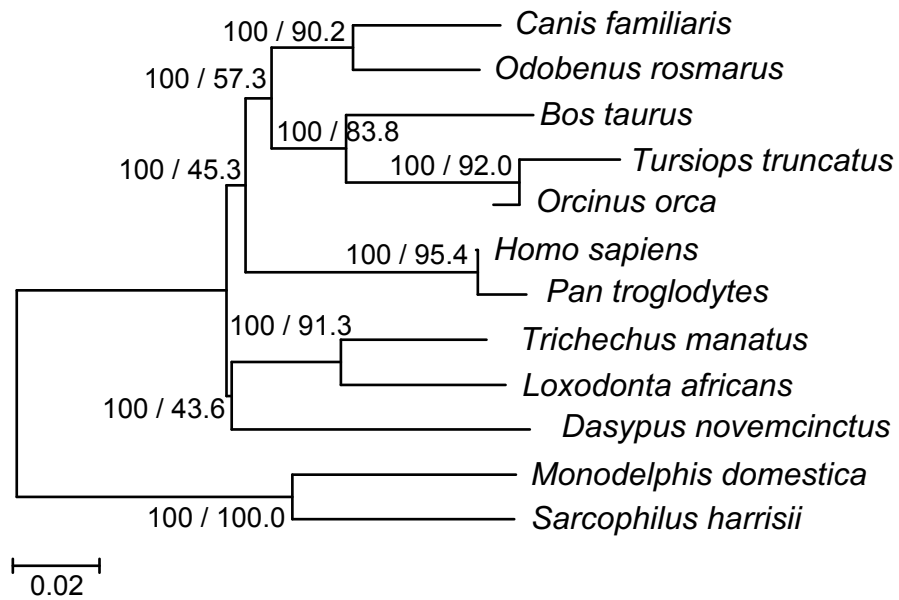

Supplementary fig. S1A.

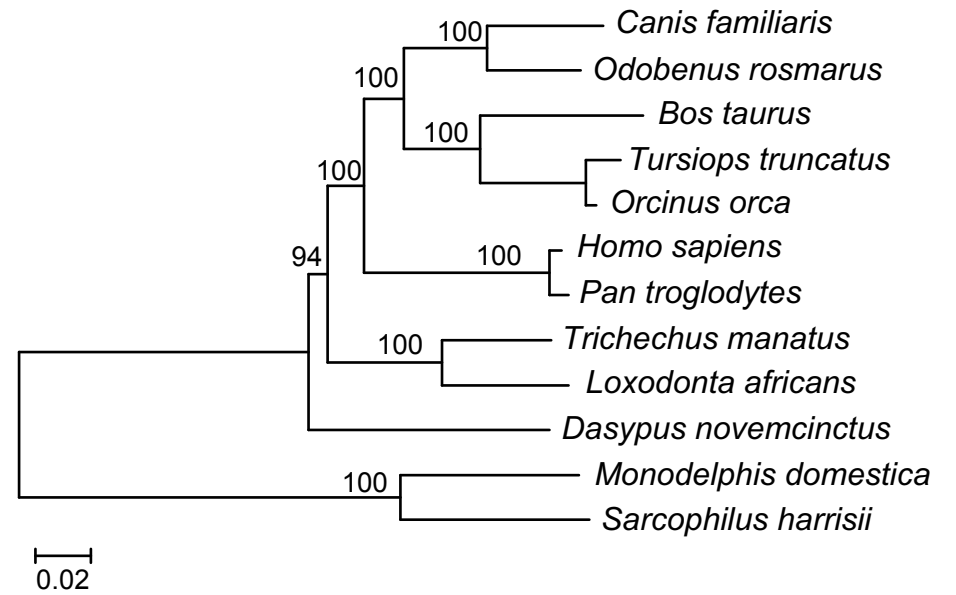

Supplementary fig. S1B.

Supplement: Supplementary Data [file supp_evw005_suppl_data.zip › SupplementaryFig1.pdf]
